# Lectin-Like Oxidized LDL Receptor-1 Is an Enhancer of Tumor Angiogenesis in Human Prostate Cancer Cells

**DOI:** 10.1371/journal.pone.0106219

**Published:** 2014-08-29

**Authors:** Iván González-Chavarría, Rita P. Cerro, Natalie P. Parra, Felipe A. Sandoval, Felipe A. Zuñiga, Valeska A. Omazábal, Liliana I. Lamperti, Silvana P. Jiménez, Edelmira A. Fernandez, Nicolas A. Gutiérrez, Federico S. Rodriguez, Sergio A. Onate, Oliberto Sánchez, Juan C. Vera, Jorge R. Toledo

**Affiliations:** 1 Biotechnology and Biopharmaceuticals Laboratory, Department of Pathophysiology, School of Biological Sciences, Universidad de Concepción, Concepción, Chile; 2 Department of Clinical Biochemistry and Immunology, School of Pharmacy, Universidad de Concepción, Concepción, Chile; 3 Department of Basic Sciences, Faculty of Medicine, Universidad Católica de la Santísima Concepción, Concepción, Chile; 4 Translational Research Unit, School of Medicine, Universidad de Concepción, Concepción, Chile; 5 Department of Pharmacology, School of Biological Sciences, Universidad de Concepción, Concepción, Chile; Duke University Medical Center, United States of America

## Abstract

Altered expression and function of lectin-like oxidized low-density lipoprotein receptor-1 (LOX-1) has been associated with several diseases such as endothelial dysfunction, atherosclerosis and obesity. In these pathologies, oxLDL/LOX-1 activates signaling pathways that promote cell proliferation, cell motility and angiogenesis. Recent studies have indicated that *olr*1 mRNA is over-expressed in stage III and IV of human prostatic adenocarcinomas. However, the function of LOX-1 in prostate cancer angiogenesis remains to be determined. Our aim was to analyze the contribution of oxLDL and LOX-1 to tumor angiogenesis using C4-2 prostate cancer cells. We analyzed the expression of pro-angiogenic molecules and angiogenesis on prostate cancer tumor xenografts, using prostate cancer cell models with overexpression or knockdown of LOX-1 receptor. Our results demonstrate that the activation of LOX-1 using oxLDL increases cell proliferation, and the expression of the pro-angiogenic molecules VEGF, MMP-2, and MMP-9 in a dose-dependent manner. Noticeably, these effects were prevented in the C4-2 prostate cancer model when LOX-1 expression was knocked down. The angiogenic effect of LOX-1 activated with oxLDL was further demonstrated using the aortic ring assay and the xenograft model of tumor growth on chorioallantoic membrane of chicken embryos. Consequently, we propose that LOX-1 activation by oxLDL is an important event that enhances tumor angiogenesis in human prostate cancer cells.

## Introduction

Lectin-like oxidized low-density lipoprotein receptor-1 (LOX-1) is a member of the scavenger receptor family [1], which mediates the recognition and internalization of oxidized LDL (ox-LDL) [2]. LOX-1 is mainly expressed in endothelial cells, although this receptor can also be found in many other cell types such as monocytes, cardiomiocytes, adipocytes, platelets, macrophages and vascular smooth muscle cells [3]. The altered expression and function of LOX-1 receptor has been associated with diseases such as endothelial dysfunction, atherosclerosis, obesity, and recently also with tumor development [4]–[6]. The activation of LOX-1 by oxLDL in human endothelial cells induces expression of adhesion molecules; pro inflammatory signaling pathways; and pro-angiogenic proteins, such as metalloproteinase-2 and 9 (MMP-2 and MMP-9), angiotensin II (Ang II) and vascular endothelial growth factor (VEGF) [7]–[10]. Angiogenesis is a physiological process, which determines the formation of new blood vessels from preexisting vessels [11]. It is considered a normal phenomenon during embryonic development, growth of the organism and wound healing [12]. However, the angiogenesis is also a fundamental process for the development and progression of several types of tumors [13]. Tumor angiogenesis is regulated by the balance between pro- and anti-angiogenic molecules released by tumor cells and tumor stromal cells such as fibroblasts and macrophages, which determine the switch-on of tumor angiogenesis [14], [15]. The gene expression in tumor angiogenesis is further regulated in response to several stimuli, including hypoxia, oxidative stress and inflammation. In this connection between stimuli and pro-angiogenic protein expression, VEGF has been identified as a major factor in tumor angiogenesis [16], [17]. Hypoxia, cytokine secretion and oxidative stress in tumor cells increase the expression of VEGF, thus inducing tumor angiogenesis [18].

LOX-1 has been suggested as a possible link between obesity, dyslipidemia, and cancer [19]. Consistently, clinical conditions of obesity and atherosclerosis have been associated to tumor progression and metastasis in prostate cancer [20]–[22]. Patients diagnosed with metabolic syndrome, chronic inflammatory diseases and autoimmune conditions showed a high incidence and aggressiveness in tumor development [23]. Indeed, recent studies have shown an increased expression of LOX-1 in human prostate adenocarcinomas at stages III and IV [5], which require new vascularization and angiogenesis for local invasion. However, the specific effect of LOX-1 in tumor angiogenesis has not yet been described.

This work demonstrates that LOX-1 and its activation using oxLDL induce tumor angiogenesis and stimulate cell proliferation in prostate cancer cells. Specifically, we demonstrate that the activation of LOX-1 using oxLDL promotes cell proliferation, and the expression of pro-angiogenic molecules VEGF, MMP-2, and MMP-9 in a dose-dependent manner. Notably, these effects were prevented in the C4-2 prostate cancer model when LOX-1 expression was knocked down. Furthermore, the angiogenic effect of LOX-1 activated by oxLDL was demonstrated using the aortic ring assay and the xenograft model of tumor growth on chorioallantoic membranes (CAM) of chicken embryos. Therefore, we propose that LOX-1 activation by oxLDL is a relevant activation pathway required for the angiogenic enhancement of tumor development of human prostate cancer cells.

## Results

### Generation of prostate cancer cell lines overexpressing LOX-1 and shRNA against olr1

**Figure 1:**
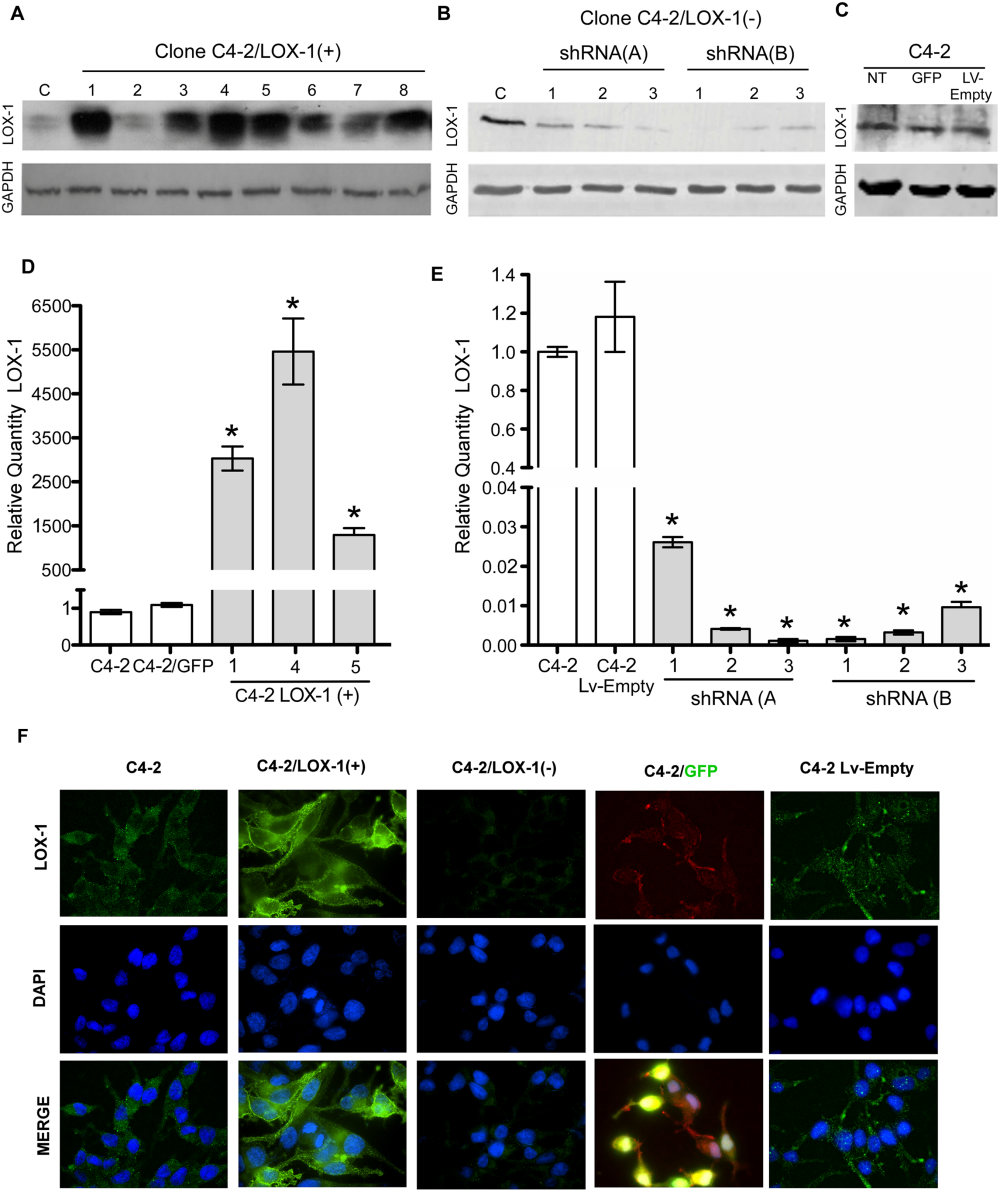
Generation of stable prostate cancer cell lines with LOX-1 over-expression and shRNA against *olr1*. **A)** Western blot for LOX-1 (40 kDa) expression in human CaP clones with overexpression of LOX-1. **B)** Western blot for LOX-1 (40 kDa) expression in human prostate cancer cell clones with LOX-1 knockdown **C)** Real-time PCR for LOX-1 expression in three clones with overexpression of LOX-1. **D)** Real-time PCR for LOX-1 expression was determined in three clones that express shRNA/LOX-1(A), and three clones that express shRNA/LOX-1(B). The data represent the means ± S.D. of three independent experiments performed in triplicate, and statistically analyzed using one-way analysis of variance and Dunnett’s post-test; (***p≤0.001, **p≤0.01, *p≤0.05).

The C4-2 prostate cancer cell lines were transduced with the lentiviral expression vector LvCW-LOX-1, encoding the *olr1* gene under the control of the cytomegalovirus promoter (CMVP), and isolated using the limiting dilution cloning method. Overexpression of LOX-1 in C4-2 cells was determined using real-time PCR, immunoblotting, and immunofluorescence. Seven different clones with LOX-1 overexpression were obtained, out of these, 3 clones were selected after Western blot experiments and analyzed using real-time PCR (Fig. 1A and D). The selected cell clone C4-2/LOX-1(+) (clone 5) had an 1.2×10^3^ fold expression over basal LOX-1 mRNA levels of the native C4-2 cell line or the C4-2/GFP overexpression control. In addition, LOX-1 overexpression in this clone was immunolocalized in the cell membrane using confocal microscopy (Fig. 1F). To determine whether overexpression mediated by lentiviral particles had an effect on the expression of LOX-1, an overexpression control (C4-2/GFP cells) was generated. We did not observed significant changes in the expression of LOX-1 in this control compared with the native C4-2 cell line (Fig. 1C and F).

The LOX-1 knockdown in C4-2 cells was achieved using a shRNA against the mRNA encoded by the *olr1* gene. C4-2 cells were transduced with lentiviral vectors for the expression of each shRNA (LvW-U6/*olr*1) sequence against *olr1* under the control of the U6 promoter. The transduced cells were isolated using the limiting dilution cloning method, and LOX-1 down-expression was analyzed using real-time PCR, immunoblotting, and immunofluorescence (Fig. 1). Two different shRNA sequences, shRNA-A and shRNA-B, were analyzed. Three different clones of shRNA-A and shRNA-B LOX-1 knockdown were obtained. The selected shRNA-B clone decreased LOX-1 expression by 98%, compared with basal LOX-1 mRNA levels of the native C4-2 cell line or the C4-2/LvEmpty knockdown control cell line (Fig. 1B–E). Furthermore, the down-expression of LOX-1 in this clone was verified by immunohistochemistry (Fig. 1F). To determine whether the knockdown mediated by lentiviral particles had an effect on the expression of LOX-1 a knockdown control (C4-2/LvEmpty) was generated. We did not observe significant changes in the expression of LOX-1 in this control compared with the native C4-2 cell line (Fig. 1C and F).

The prostate cancer cell models obtained, namely C4-2/LOX-1(+) [clone 5], C4-2/LOX-1(−) [clone shRNA-B1] and native C4-2 cell line, were used as models in all assays performed in this work.

### oxLDL has not cytotoxicity effects in prostate cancer cell models

**Figure 2:**
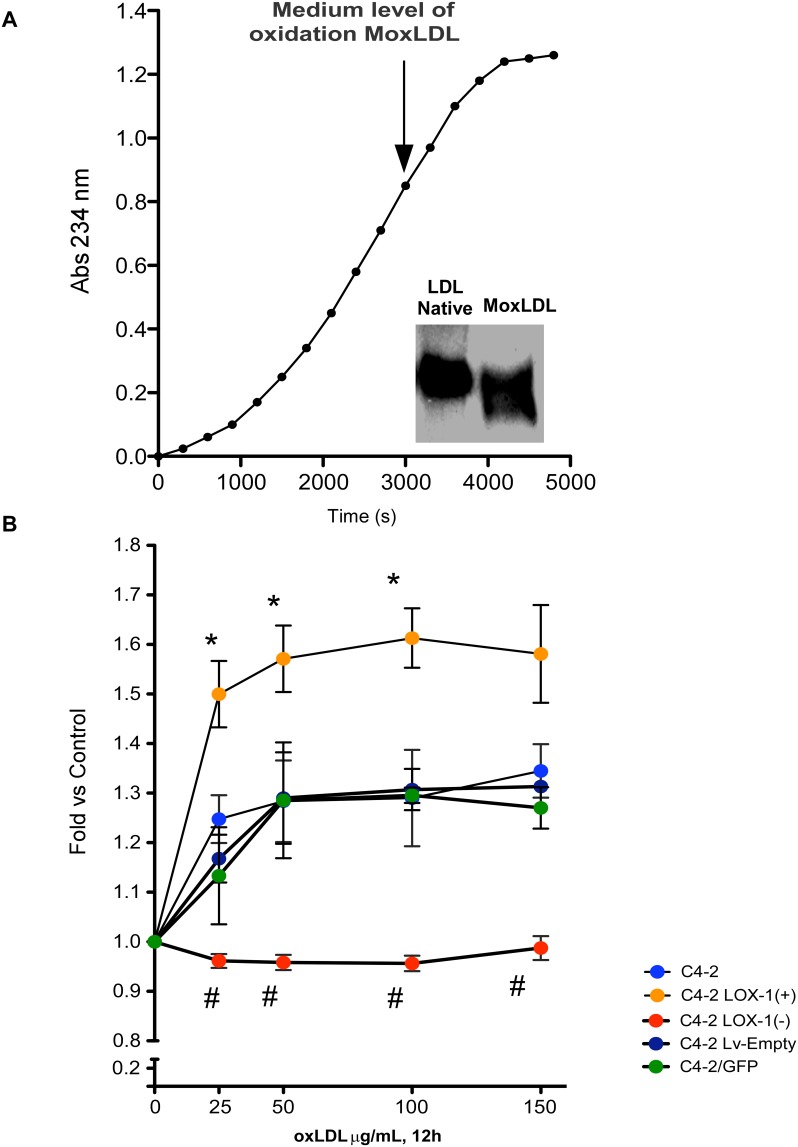
oxLDL characterization and citotoxicity assay. **A)** oxLDL obtained from human plasma was oxidized with 7 uM of CuSO_4_, monitored by spectrophotometry at λ 243 nm and electrophoresed in 1% agarose gels with sodium borate buffer. **B)** Cytotoxicity assay of prostate cancer cell models treated with 25, 50, and 100 µg/mL oxLDL during 12 hours. The data represent the means ± S.D. of three independent experiments performed in triplicate and statistically analyzed using one-way ANOVA and Dunnett’s post-test (* or # *p≤0.05*).

Starting from the oxidation of native LDL from normolipemic patient we obtained a fraction of medium level oxidized LDL (oxLDL) which was checked by generation of conjugated dienes and sodium borate buffer electrophoresis in 1% agarose (Fig. 2A). To determine whether the oxLDL obtained had some cytotoxic effect we incubated the prostate cancer cell models with oxLDL (25 to 150 µg/mL) during 12 hours. Our results showed no cytotoxic effects of oxLDL on any of the prostate cell models for the range of concentrations assayed (Fig. 2B). However, we observed a significant increase in cell proliferation of C4-2, C4-2/GFP, C4-2/LvEmpty and C4-2/LOX-1(+) cellular models for all oxLDL concentrations used, compared to the same untreated prostate cancer cell models. Moreover, a significant increase in cell proliferation was observed in C4-2/LOX-1(+) compared with the C4-2, or the overexpression and knockdown controls for all oxLDL concentrations analyzed. However, the proliferative effect was totally prevented in the C4-2/LOX-1(−) cell model, over all concentrations analyzed (Fig. 2B).

### The oxLDL ligand increases the expression of pro-angiogenic markers

**Figure 3:**
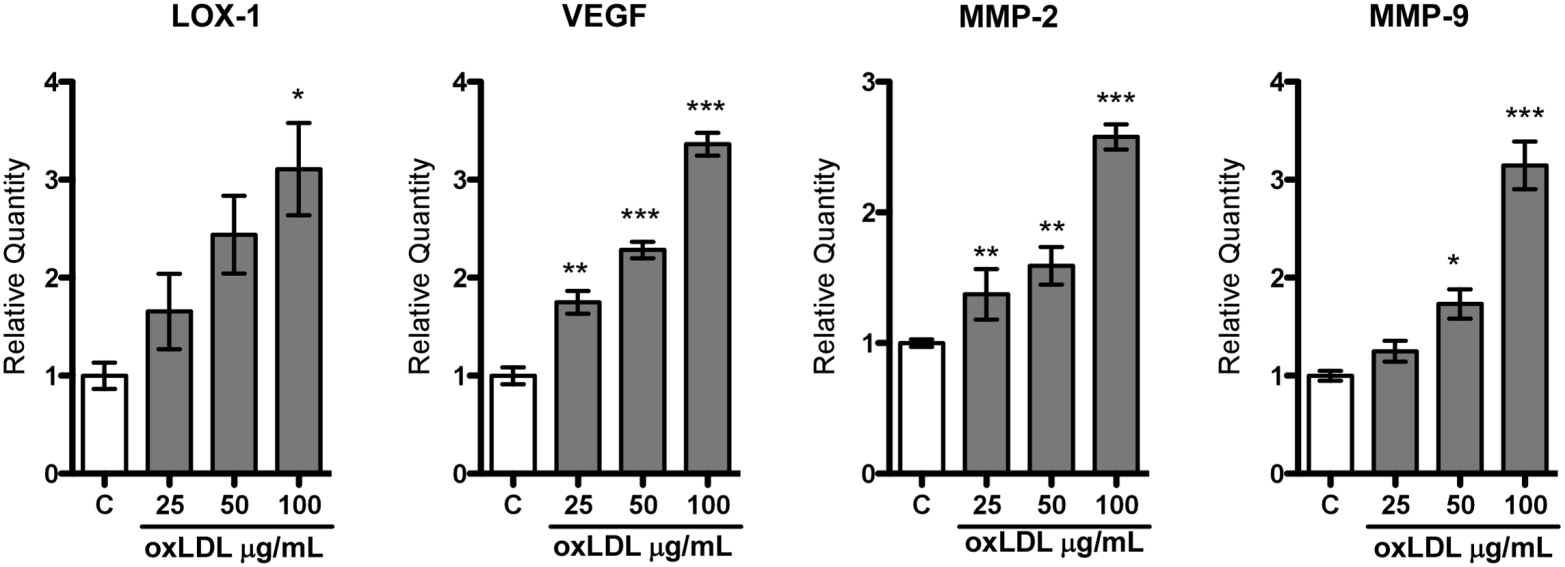
The oxLDL ligand increases the expression of pro-angiogenic markers. Relative quantification of LOX-1, VEGF, MMP-2, and MMP-9 expression was performed using real-time PCR in human prostate cancer cell line C4-2 incubated with increasing concentration of oxLDL (25, 50, 100 µg/mL) for 12 hours. The data represent mean ± S.D. of three independent experiments performed in triplicate, and statistically analyzed using one-way analysis of variance and Dunnet post-test; (***p≤0.001, **p≤0.01, *p≤0.05).

The prostate cancer C4-2 cell line was incubated with increasing concentrations of oxLDL (25, 50, 100 µg/mL) during 12 hours, and the expression of the pro-angiogenic markers VEGF, MMP-2 and MMP-9 was analyzed using real-time PCR. Our results showed a significant increase in the expression of VEGF, MMP-2 and MMP-9, proportional to the oxLDL concentrations used, with a respective 3.5-, 2.5-, and 3-fold increase, when 100 µg/mL of oxLDL was used. Moreover, LOX-1 expression was also proportionally increased with the concentrations of oxLDL used, showing a 3-fold increase at 100 µg/mL (Fig. 3).

### Increased expression of pro-angiogenic markers in prostate cancer cells requires activation of LOX-1 by oxLDL

**Figure 4:**
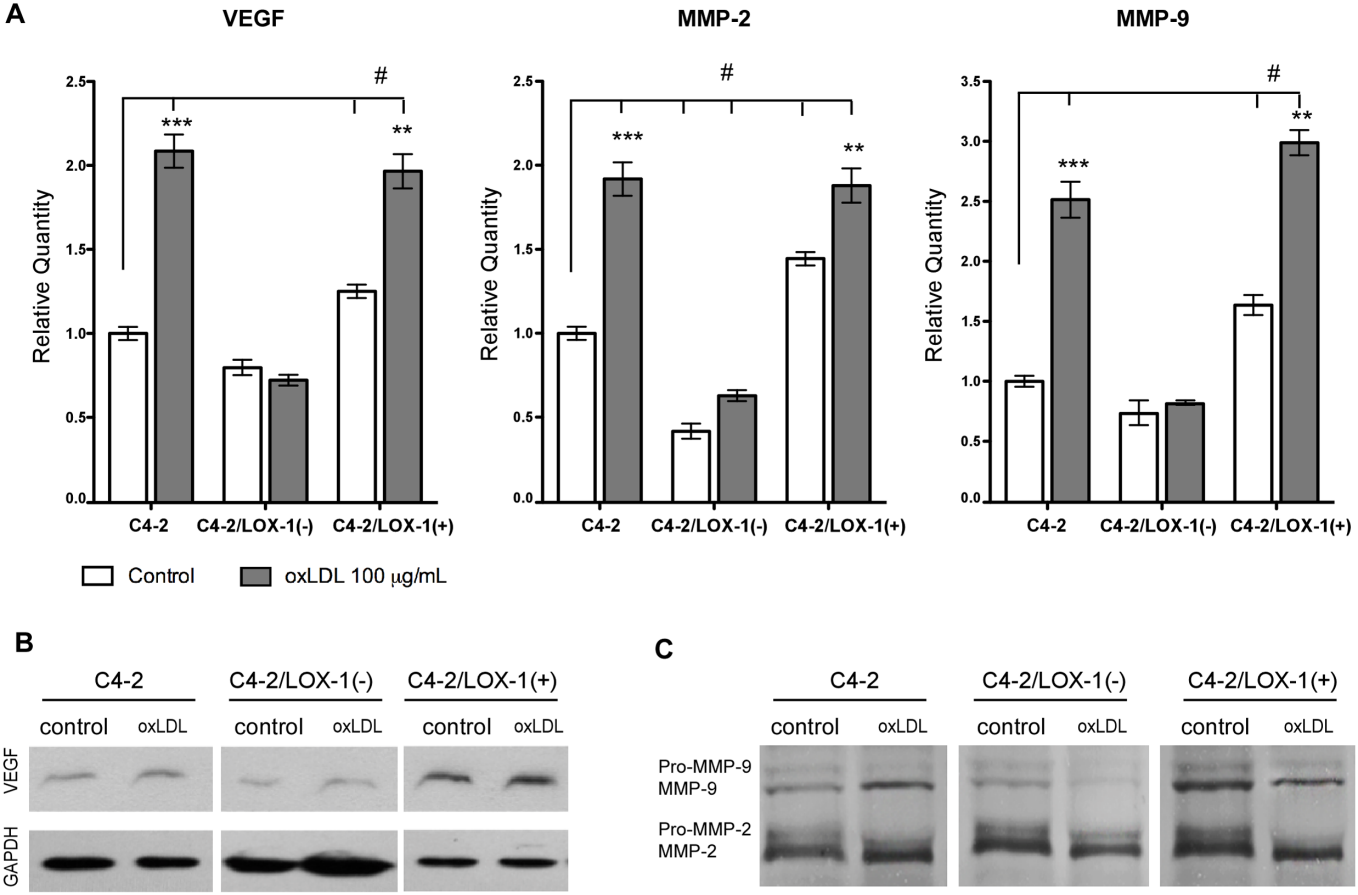
Activation of LOX-1 by oxLDL increases the expression of pro-angiogenic markers. C4-2, C4-2/LOX-1(−), and C4-2 LOX-1(+) human prostate cancer cell models were incubated in the with or without oxLDL [100 µg/mL] for 12 hours. **A)** Relative quantification of VEGF, MMP-2, and MMP-9 expression was analyzed using real-time PCR. The data represent the means ± S.D. of three independent experiments performed in triplicate and statistically analyzed using one-way analysis of variance and Dunnet post-test; (***p≤0.001, **p≤0.01, *p≤0.05). **B)** Western blot for VEGF expression (30 kDa). **C)** Zymogram for MMP-2 (72 kDa pro-form; 64 kDa active-form) and MMP-9 (92 kDa pro-form; 84 kDa active-form) activities in conditioned medium.

The prostate cancer cells models C4-2/LOX-1(−) and C4-2/LOX-1(+) were incubated with 100 µg/mL oxLDL during 12 hours, and expression of the pro-angiogenic markers (VEGF, MMP-2 and, MMP-9) was analyzed. Expression of VEGF, MMP-2 and, MMP-9 in the C4-2 cell line incubated with oxLDL increased significantly (2-, 2-, and 2.5-fold, respectively), compared with untreated C4-2 cells (Fig. 4A). Interestingly, the expression of pro-angiogenic markers induced by oxLDL, was prevented in the C4-2/LOX-1(−) prostate cancer cell model. On the other hand, the stimulation of C4-2/LOX-1(+) with oxLDL significantly increased the expression of all pro-angiogenic markers analyzed (VEGF and MMP-2, 2-fold; MMP-9 3-fold), compared with untreated C4-2 cells. The expression of VEGF, MMP-2, and MMP-9 in C4-2/LOX-1(+) cells was also significantly increased (1.6-, 1.5-, and 1.4-fold, respectively), even in the absence of oxLDL stimulation, and decreased by 20%, 50%, and 20% in C4-2/LOX-1(−) cells, compared with the endogenous expression in the C4-2 cell line without oxLDL stimulation (Fig. 4A).

The conditioned media from prostate cancer cell models incubated with oxLDL [100 µg/mL] were collected and concentrated. Later VEGF expression was evaluated by immunoblotting, and the activity of MMP-2 and MMP-9 was analyzed by zymography. Our results indicted that VEGF expression was increased in C4-2 cells incubated with oxLDL, when comparing with C4-2 cells without oxLDL, whereas the expression of VEGF was prevented in C4-2/LOX-1(–) cells incubated with oxLDL [100 µg/mL]. Moreover, VEGF expression was increased in C4-2/LOX-1(+) cells with o without oxLDL treatment (Fig. 3B). Analysis of metalloproteinase activity showed an increment in the gelatinase activity of MMP-2 and MMP-9 in the conditioned medium from C4-2 cells incubated with oxLDL, and was prevented in conditioned medium from C4-2/LOX-1(–) cells incubated with oxLDL. Furthermore, LOX-1 overexpression in C4-2/LOX-1(+) cells showed an increased activity of MMP-2 and MMP-9 with or without oxLDL stimulation (Fig. 4C).

### Activation of LOX-1 by oxLDL promotes the generation of sprout in mouse aortic rings

We analyzed C4-2 model cells incubated with oxLDL to determine whether the generation of endothelial sprouts could be stimulated in the aortic ring assay by the differential secretion of pro-angiogenic markers. The C4-2, C4-2/LOX-1(–) and C4-2/LOX-1(+) cell models were incubated with 100 µg/mL of oxLDL during 12 hours, and the conditioned media was collected, concentrated and used for the treatment of mouse aortic rings.

**Figure 5:**
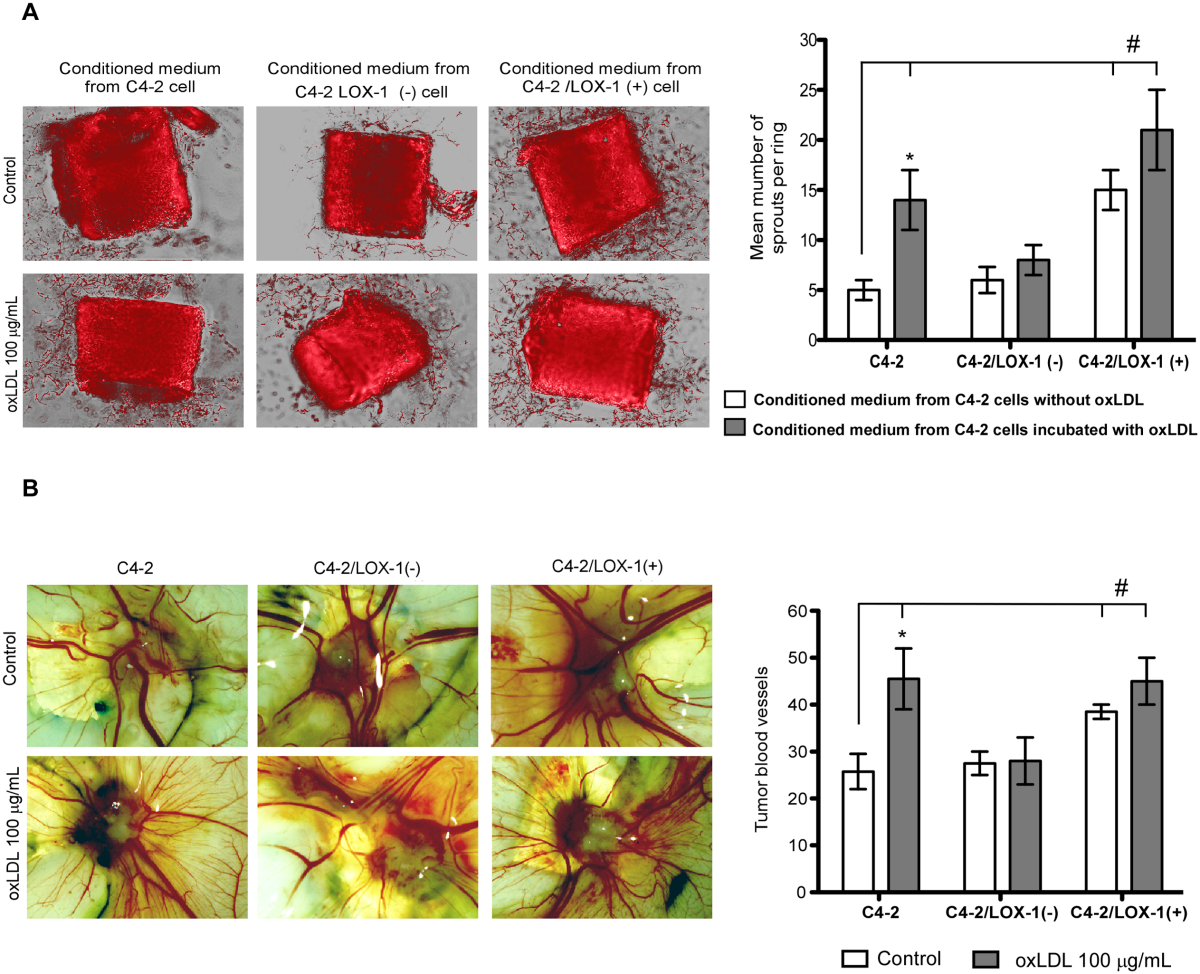
The activation of LOX-1 using oxLDL promotes the generation of sprouts in mouse aortic ring assays. **A).** Microphotography of mouse aortic rings and quantification of sprouts per ring incubated with conditioned medium from prostate cancer C4-2, C4-2/LOX-1(–), and C4-2 LOX-1(+) cells previously stimulated with or without oxLDL [100 µg/mL]. **B)** LOX-1 activation by oxLDL promotes tumor angiogenesis in CaP xenograft models on chorioallantoic membrane of chicken embryos. Microphotography and quantification of tumor blood vessels in xenografts of C4-2, C4-2/LOX-1(–), and C4-2 LOX-1(+) cells, previously incubated with or without oxLDL [100 µg/mL], in chorioallantoic membranes of 10-days-old chicken embryos. The data represent the means ± S.D. of three independent experiments performed in triplicate, and was statistically analyzed using one-way analysis of variance with Dunnett’s post-test (***p≤0.001, **p≤0.01, *p≤0.05).

The stimulation of aortic rings with conditioned media from C4-2 and C4-2/LOX-1(+) cells treated with oxLDL significantly enhanced the generation of sprouts compared to the untreated C4-2 cell line. However, the differences between aortic ring sprout generation using conditioned media from C4-2/LOX-1(–) with or without oxLDL stimulation, were not significant. Thus, over-expression of LOX-1 promoted sprouts generation with or without oxLDL stimulation (Fig. 5A).

### Activation of LOX-1 by oxLDL promotes angiogenesis in prostate cancer cell xenografts on chorioallantoic membrane of chicken embryos

For angiogenesis studies on chorioallantoic membrane (CAM) of chicken embryos, prostate cancer cell clones C4-2, C4-2/LOX-1(−), and C4-2 LOX-1(+) were pre-incubated with oxLDL 100 µg/mL for 2 hours. Approximately 1×10^6^ cells were then inoculated on CAM of 10-days-old chicken embryos. Five days after inoculation, tumors were extracted from the CAMs and the extent of tumor vascularization was analyzed. C4-2 cells pre-incubated with oxLDL showed a significant increase of tumor vascularization compared with the untreated C4-2 cells. Further, LOX-1 overexpression in C4-2/LOX-1(+) cells also showed an increased tumor vascularization when the cells were incubated with or without oxLDL compared to the untreated C4-2 cells. Notably, C4-2/LOX-1(–) cells treated with oxLDL did not show significant variations in tumor vascularization, compared with both C4-2 and C4-2/LOX-1(–) untreated cells (Fig. 5B).

### The *olr1* gene is overexpressed in public array datasets from metastatic prostate cancer

**Figure 6:**
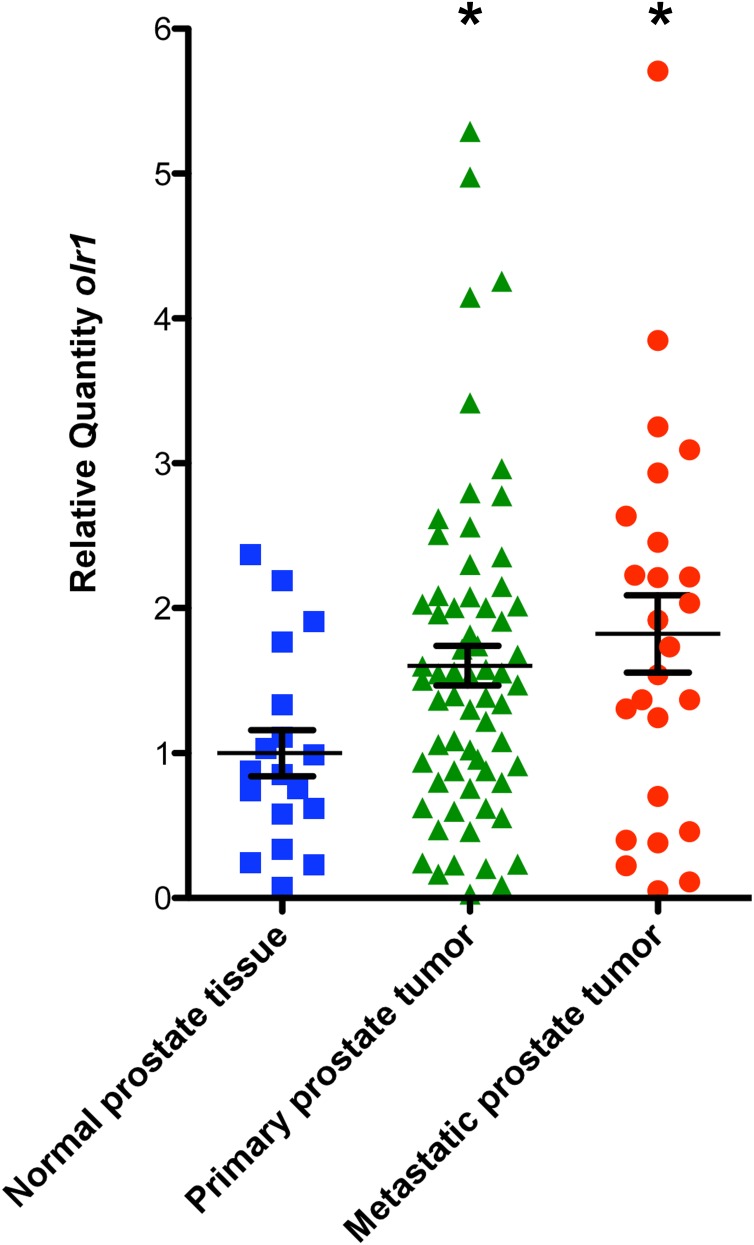
Analysis of *olr1* expression in prostate cancer progression using public databases arrays. The expression of *olr1* at different stages of prostate cancer tumor progression was determined using the public database GDS2545 at the NCBI Gene Expression Omnibus (GEO). The data represent the means ± S.E. of human normal donor tissue n = 17, primary prostate tumors n = 64 and metastatic prostate tumors n = 50, which were statistically analyzed using a *t* test (*p≤0.05).

With the aim to determine if the observed effects on tumor angiogenesis mediated by oxLDL/LOX-1 in C4-2 prostate cancer cells had any clinical relevance in patients, we used NCBI Gene Expression Omnibus (GEO) database to determine the expression of *olr1* receptor in the GDS2545 dataset, which has different stages of prostate cancer progression. This data allows the comparative analysis of human metastatic prostate tumors, primary prostate tumors and normal donor tissue. In this context, the expression of *olr-1* was found to be significantly increased in primary prostate tumors and metastatic prostate tumors compared with normal prostate tissue (Fig. 6).

## Discussion

In this work we demonstrate, for the first time, the role of the LOX-1 receptor and its activation using oxLDL, on the proliferation and angiogenesis of human C4-2 prostate cancer cells. Specifically, we demonstrate that an increase in oxLDL concentrations promoted cell proliferation and significantly and proportionally increases the expression of the pro-angiogenic markers VEGF, and the metalloproteinases MMP-2 and MMP-9. In addition, cellular stimulation of LOX-1 with oxLDL also increased the expression of the LOX-1 receptor, generating a positive feedback-loop of LOX-1 expression.

The association of LOX-1 and oxLDL with diseases, such as endothelial dysfunction, atherosclerosis, acute myocardial infarction, stroke, diabetes, hypertension, metabolic syndrome and obesity, has been extensively studied [3], [5], [24]. Obesity, itself, has also been associated as an important risk factor for atherosclerosis, type 2 diabetes mellitus and cancer, as well as other diseases [25]. Clinical studies suggest an association between high serum oxLDL concentrations and an increased risk of developing colon cancer [26]. Furthermore, oxLDL concentrations have also been shown to increase in obese patients [27].

Consistent with these findings, previous reports had shown that obesity is associated with an increased risk for developing highly malignant and metastatic prostate cancer [21], [28]. Moreover, in the Transgenic Adenocarcinoma of the Mouse Prostate (TRAMP) mouse model, feeding of the mice with an hypercaloric diet, increases the histological grade of the prostate tumors, the number of metastases, tumor mass, and the degree of vascularization, as compared to TRAMP mice fed with normal diet [29].

There is a special relevance in the relationship between cholesterol and prostate cancer, because androgen and other steroid hormones are synthesized from cholesterol, and are fundamental in the development and maintenance of prostate cancer [30]. Under physiological conditions androgens are involved in the acquisition of secondary sexual characteristics, growth and normal development of the prostate. However, they have also been associated with tumor progression in prostate cancer [31]. Thus, many of the prostate cancers refractory to androgen deprivation, increase cholesterol uptake, which is the primary substrate for androgen synthesis [31], [32]. In this respect, the major source of cholesterol for tumor cells is LDL, which is mainly endocyted by the LDL receptors (LDLR) [33], [34]. However, we consider that the oxidant and pro-inflammatory tumor microenvironment rich in ROS [35] could be promoting LDL modification by lipoperoxidation [36], avoiding the recognition by LDLR and favoring its uptake by scavenger receptors of oxLDL. According to this, we analyzed the expression of scavengers receptors for modified lipoproteins and the expression of the LDL receptor in the GDS2545 dataset, obtained from the public database (GEO profile) [37]. Therein, we observed a significant increase in the expression of the scavenger receptors *olr1, scarf* and *scarb1* in primary tumors and metastasis of prostate cancer, and a significant increase in the expression of the *CD36* and *olr1* receptor in prostate cancer metastasis compared to normal prostate tissue. Interestingly, the expression of LDLR showed a significant decrease in its expression in tumor metastasis and no significant difference was observed between primary prostate tumors and normal prostate tissue (data not shown).

Considering this, high concentrations of circulating LDL in obese patients with prostate cancer could contribute to tumor progression through LOX-1 activation by oxidation modified LDL (oxLDL) generated in the oxidant microenvironment, present in the prostate tumor stroma. In this respect, we generated a medium level oxidized LDL, which should be representative of the oxLDL present in the tumor microenvironment. Our results demonstrated that the oxLDL used in this study has no cytotoxic effect in any of the C4-2 prostate cancer cells models assayed. In this regard, it has been reported that oxLDL has a differential cytotoxic effect depending on the cell type. Cancer cells lines K562/AO2 (Leukemia) and EC9706 (esophageal carcinoma) treated with oxLDL present a higher rate of cell viability compared whit non-tumor cells such as HUVEC (Human umbilical vein endothelial cells) [38].

Interestingly, we observed a significant increase in cell proliferation of the C4-2, C4-2/GFP, C4-2/LvEmpty and C4-2/LOX-1(+) cellular models, over the whole range of oxLDL concentrations assayed compared to the untreated cell lines. Also, the C4-2/LOX-1(+) cell model showed a higher proliferation compared with the control cells models C4-2, C4-2/GFP, and C4-2/LvEmpty, for all concentrations assayed. However, the proliferative effect of oxLDL was prevented in the C4-2/LOX-1(−) cell model, indicating that this effect is closely related to the activation of the LOX-1 receptor by oxLDL. In this sense, the proliferative effect promoted by oxLDL/LOX-1 has been described in muscle cells, endothelial cells and recently in breast cancer cells through activation pathways that involve p38 (MAPK), p44/42 MAPK, and NF- kB [6], [39]. However, the proliferative effects on prostate cancer cells had not yet been described.

*In vitro* studies have shown that oxLDL stimulation of human umbilical vein endothelial cells (HUVECs) activates the expression of the LOX-1 receptor, generating an overexpression of adhesion molecules, inflammatory proteins, and the MMP-2 and MMP-9 metalloproteinases, [8], [40], [41]. Furthermore, endothelial cells from bovine aorta treated with ox-LDL promote capillary formation regulated by LOX-1, and the activation of NADPH oxidase/MAPKinase/NF-kappa B, with an increase in VEGF expression [9], [42]. Our results demonstrated that the expression of VEGF, and the activation of MMP-2 and MMP-9, is closely mediated by oxLDL/LOX-1 in C4-2 prostate cancer cell line.

We also confirm, using the aortic ring assay and the chicken embryo CAM xenograft model, that the increase of VEGF, MMP-2 and MMP-9 expression mediated by LOX-1/oxLDL has a tumoral angiogenic effect both *in vivo* and *ex vivo* on C4-2 prostate cancer cells models. This is consistent with previous *ex vivo* studies, where pro-angiogenic factors like angiotensin II did not induce capillary sprout formation in aortic rings isolated from *olr-1 KO* mice [10]. Likewise, the ablation of the *olr-1* gene markedly decreased the choroidal neovascularization in murine models of laser-induced injury [43]. Signaling induced by oxLDL/LOX-1 could have a critical role in the process of tumor angiogenesis because microvessel density is an important prognostic indicator in prostate cancer, and is associated with clinical stage, progression, metastasis, and prostate cancer survival [44], [45].

In conclusion, we demonstrate a direct relationship between obesity factors, such as LOX-1/oxLDL and the enhancement of expression of proliferation and pro-angiogenic markers, which have a biological effect in tumor angiogenesis *ex vivo* and *in vivo* in C4-2 prostate cancer cells models. Hence, we suggest that LOX-1 could be an essential regulator in prostate cancer cells, with the ability to enhance tumor angiogenesis towards malignancy and metastasis in obese patients.

## Materials and Methods

### Cell culture

Human C4-2 prostate cancer [46], [47] and HEK-293 FT cells were grown in RPMI 1640 and DMEM medium respectively, supplemented with 2 mM of L-glutamine (Hyclone), 10% fetal bovine serum (FBS) and 1% penicillin streptomycin (GIBCO).

### Animals

Male BALB/c mice were obtained from Harlan Winkelmann GmbH (Borchen, Germany) and housed under controlled and sterile ambient conditions. Four mice (8–12 weeks old) were used for the experiments.

Eggs of *Gallus gallus domesticus* were obtained from ISP (Instituto de Salud Pública, Chile), were washed with a 7 µM copper sulfate solution to prevent fungal contamination, and were incubated at 37.5°C and 80% of relative humidity for 10 days. The eggs were flipped periodically with an automatic dual turner (GQF Hova-bator Manufacturing Co. USA) to prevent adhesion and rupture of the CAM from the eggshell. Embryos inoculated with the C4-2 cells models were incubated at 37.5°C, 80% humidity and 5% CO_2_ for 5 days.

All the studies involving experimentation with animals were in accordance with the guidelines and recommendations of the NIH Guide for the Care and Use of Laboratory Animals (current edition) and followed the policies indicated in the Chilean Biosafety Manual of Conicyt (National Agency for Science and Technology). The experimental protocols were drafted by the authors and approved by the Institutional Ethics Committee. In all cases, supervision of veterinary authorities from the School of Biological Sciences, Universidad de Concepción, Chile, was guaranteed. When mice were used for experimentation, they were housed in individual rooms and appropriate feeding, water supply and health monitoring was permanently provided. Animals euthanized were humanly handled. They were subjected to initial anesthetization and potassium chloride injection intravenously to avoid suffering.

### ox-LDL preparation

Blood samples (20 mL) were obtained from four volunteers, who signed a written consent. This protocol was approved by Ethics Committee of the Universidad de Concepción, Chile. Human LDL (1.019 to 1.063 g/mL) was isolated from the blood plasma of healthy human subjects by sequential ultracentrifugation at 4°C. LDL was dialyzed against filtered (0.45-µm) LDL buffer (150 mmol/L NaCl; 0.24 mmol/L EDTA; pH 7.4), changed thrice, for 36 hours at 4°C. After extensive dialysis against PBS, with 3 changes, for 36 hours at 4°C, oxidized LDL was prepared by incubating purified LDL with 7 µM CuSO_4_ for 3 hours at 37°C. Modification of LDL was monitored by spectrophotometry through the generation of conjugated dienes at λ 243 nm, and then an electrophoresis in 1% agarose gel in sodium borate buffer was realized to determine the change in the electrophoretic migration of oxLDL compared with LDL. The gel was stained with 0.25% Coomassie Blue R-250, during 2 hours, and destained during 4 hours using 5% MeOH, 7.5% HOAC, 87.5% H_2_O. The bands of LDL and oxLDL were analyzed with the Odyssey CLX instrument (LI-COR, EEUU).

### Cytotoxicity evaluation

The prostate cancer cell models were incubated with 25, 50, and 100 µg/mL oxLDL during 12 hours. After that, the medium was removed, the cultures were washed with phosphate-buffered saline, and cell viability was measured incubating the cultures with 10 mg/ml of 3-(4,5-dimethylthiazol-2-yl)-2,5-diphenyl tetrazolium bromide (MTT) at 37°C for 30 min, and measuring absorbance at 570 nm in solubilized cells using a UV-visible SPECTROstar *Nano* spectrometer (BMG LABTECH, Germany).

### Lentiviral vectors that express orl-1 or shRNA against olr1

The human LOX-1 sequence was sub-cloned into the lentiviral pLCW vector, generating the pLCW-LOX-1 construct. Further, a sequence coding for GFP was inserted in the pLCW vector, generating the pLGW plasmid.

To generate the pLU6W plasmid, the cytomegalovirus promoter (CMVP) was removed from the pLCW lentiviral vector, and in its place, an U6 promoter obtained from the retroviral plasmid pGFP-V-RS was inserted. The codifying shRNA sequences were purchased as oligos of 65 bases (IDT, Coralville, USA) and phosphorylated with polynucleotide kinase PNK (New Englad Biolabs, UK). For annealing, oligos were incubated at 95°C for 5 minutes, and left at 25°C during 1 hour. Hybridization of the shRNAs was analyzed by electrophoresis in 2% agarose gels. The double strand codifying shRNA sequences were cloned using *Xma*I and *Xho*I restriction sites into pLU6W, generating the pLU6W/shRNAolr1 (A and B variants).

### Lentiviral vector production

Production of lentiviral particles was performed by co-transfection of HEK-293FT cells with the constructs generated (pLCW-LOX-1, pLGW, pLU6W or pLU6W/shRNA*olr*1,) and three helper plasmids: pLP1, pLP2 and pLP/VSVG (ViraPowerTM Lentiviral Expression System, Invitrogen-Life Technologies, USA). Forty-eight hours post-transfection, the culture media were collected, centrifuged at 3000 rpm for 10 minutes, and the supernatants filtered through 0.45 µm pore size membranes (Millipore). Later, the lentiviral particles (Lv-LOX-1, Lv-GFP, Lv-shRNA/*olr1* and Lv-Empty [derived from pLU6W plasmid]) were concentrated by ultracentrifugation at 25000 rpm (SW28 rotor) for 90 minutes. The supernatant was removed and the pellet was resuspended in 100 µL of culture medium. Finally, the lentiviral particles were collected, aliquoted and stored at −80°C until used.

### Stable cell line generation for over-expression or knockdown of LOX-1

C4-2 cells were seeded into 96-well plates and cultured up to 80% confluence. Then, the cells were incubated with the lentiviral vectors Lv-LOX-1, Lv-GFP, Lv-shRNA/*olr1* or Lv-Empty, during 12 hours. After incubation, the culture medium was removed, and fresh RPMI medium supplemented with 10% FBS was added to the cells, which were cultured until confluence. The transduced cells, with each of the lentiviral vectors, were seeded into 96 wells by serial micro-dilution down to 1 cell per well. The cultures were maintained with RPMI-10% FBS to reach 100% confluence. Stable cell pools were established for [C4-2/LOX-1(+), C4-2/GFP, C4-2/LOX-1(–) and C4-2/Lv-Empty], and LOX-1 over-expression or knockdown was verified by Western blot, real-time PCR and immunohistochemistry.

### Real-Time PCR

Total RNA was purified using TRIZOL (Sigma, USA). The PCR reaction was performed with the commercial kit KAPA SYBR FAST qPCR and the equipment for Stratagene MX3000P real-time PCR. The qPCR was performed using RNA as template, the primers were ordered from Integrated DNA Technologies (Coralville, USA), LOX-1 (sense 5′-AGATCCAGACTGTGAAGGACCAGC-3′ and antisense 5′-CAGGCACCACCATGGAGAGTAAAG-3′), VEGF (sense 5′-CTGCTCTACCTCCACCATGC-3 and antisense 5′-AGCTGCGCTGATAGACATCC-3′), MMP-2 (sense 5′-CCTATCTCAGGGTTAAAAGAGAG-3′ and antisense 5′-GCACAAACAGGTTGCAGCTC-3′), MMP9 (sense 5′-ACGCACGACGTCTTCCAGTA-3′ and antisense 5′-TTGGTCCACCTGGTTCAACTC-3′) and GAPDH (sense 5′-ACCCCTTCATTGACCTCAAC-3′ and antisense 5′-ATGACAAGCTTCCCGTTCTC-3′). The results were analyzed as CT relative quantification. The comparative threshold cycles values were normalized for GAPDH reference gene.

### Immunodetection

Western blot and immunostaining were performed using standard protocols. Anti-LOX-1 polyclonal antibody was purchased from R&D Systems Inc. (Minneapolis, MN, USA), anti-VEGF and anti-GAPDH antibodies were obtained from Santa Cruz Biotechnology (Santa Cruz, CA, USA). Anti-mouse IgG/HPR and anti-goat IgG/HPR secondary antibodies, used for Western blot assays were obtained from DAKO (California, USA). Anti-goat IgG/TEXAS-RED and anti-goat IgG/FITC secondary antibodies, used for immunofluorescence were also obtained from DAKO (California, USA).

### Aortic ring assays

Conditioned medium obtained from C4-2, C4-2/LOX-1(+), and C4-2/LOX-1(–) cell models incubated with or without oxLDL [100 µg/mL] for 12 hours were collected and concentrated 10 fold. Mouse aortic rings were grown on type I collagen matrix, incubated with a mix of Opti-MEM culture medium and concentrated conditioned medium in a 10∶1 ratio. Opti-MEM/conditioned medium were changed daily during 5 days. Aortic rings were then fixed with 4% paraformaldehyde, stained with phalloidin-Texas-Red and analyzed by confocal microscopy (Olympus IX81, Japan).

### Xenografts of prostate cancer cell models with overexpression or knockdown of LOX-1 on chorioallantoic membranes of chicken embryos

C4-2, C4-2/LOX-1(+), and C4-2/LOX-1(–) prostate cancer cells were pre-incubated with oxLDL [100 µg/mL] for 2 hours, and 1×10^6^ cells were inoculated on chorioallantoic membranes (CAM) of 10-days-old chicken embryos. Five days post-inoculation, the tumors were extracted from the CAM and the extent of tumor vascularization was determined by quantifying blood vessels area [48], [49], using the SZX16 Olympus stereoscope and the ImageJ software.

### Statistical analysis

Data are presented as means with standard deviations (sd). Statistical analyses were performed with Graphpad Prism v5.0 software. Multiple comparisons were analyzed by one-way ANOVA with Dunnett's post test. A p value<0.05 was considered to be significant.
